# Independent Control over Multiple Cell Types in Space and Time Using Orthogonal Blue and Red Light Switchable Cell Interactions

**DOI:** 10.1002/advs.201800446

**Published:** 2018-06-17

**Authors:** Simge G. Yüz, Julia Ricken, Seraphine V. Wegner

**Affiliations:** ^1^ Max Planck Institute of Polymer Research Ackermannweg 10 55128 Mainz Germany; ^2^ Department of Biophysical Chemistry University of Heidelberg Im Neuenheimer Feld 253 69120 Heidelberg Germany

**Keywords:** cell interaction, orthogonality, photoswitchable proteins, spatiotemporal control, wavelength selectivity

## Abstract

Independent control over multiple cell–material interactions with high spatiotemporal resolution is a key for many biomedical applications and understanding cell biology, as different cell types can perform different tasks in a multicellular context. In this study, the binding of two different cell types to materials is orthogonally controlled with blue and red light providing independent regulation in space and time. Cells expressing the photoswitchable protein cryptochrome 2 (CRY2) on cell surface bind to N‐truncated CRY‐interacting basic helix–loop–helix protein 1 (CIBN)‐immobilized substrates under blue light and cells expressing the photoswitchable protein phytochrome B (PhyB ) on cell surface bind to phytochrome interaction factor 6 (PIF6)‐immobilized substrates under red light, respectively. These light‐switchable cell interactions provide orthogonal and noninvasive control using two wavelengths of visible light. Moreover, both cell–material interactions are dynamically switched on under light and reversible in the dark. The specificity of the CRY2/CIBN and PhyB/PIF6 interactions and their response to different wavelengths of light allow selectively activating the binding of one cell type with blue and the other cell type with red light in the presence of the other cell type.

In tissues, multiple cell types work together to perform complex tasks and it is their relative arrangement that governs the exchange between different cell types. To achieve proper organization in a multicellular tissue, different cell types express different cell adhesion molecules (e.g., integrins) that bind to different ECM (extracellular matrix) components, link to the actin cytoskeleton, and activate specific intracellular signaling pathways. The spatially and temporally controlled expression of adhesion molecules, production of ECM, and activation of signaling position the different cell types to the right place at the right time.^1^ Further, the dynamic cell–matrix adhesions enable cells to rearrange within a tissue over time during processes such as wound healing and embryo development. To produce multicellular systems that match the sophistication of nature, it is crucial to be able to dynamically and independently control the binding of multiple cell types to synthetic material in space and time. Such multicellular systems would be valuable tools to study cell biology and for applications in cell sorting, biomedicine, medical implants, immunology, and cell‐based screening devices.^2^

The first challenge is the independent control of cell–material interactions of multiple cell types. This requires multiple, specific natural or artificial cell–material interactions and so far there are only a few examples that are known to achieve this. The fact that different integrins bind to similar binding motifs makes it challenging to establish multiple specific cell–material interactions based on the integrin receptors. Yet, the selective adhesion of cells that express one type of integrin has been achieved on substrates modified with either α_v_β_3_‐ or α_5_β_1_‐specific peptidomimetics.^3^ Similarly, materials have been modified with antibodies for specific cell surface markers to bind and separate subtypes of immune cells.^4^ Another strategy is to modify the surfaces of cells with synthetic orthogonal interaction partners such as single‐stranded DNAs to selectively bind these cells to substrates with complementary DNA strands.^5^ To prompt cell adhesion to specific areas on such substrates, different adhesion molecules have been immobilized to preform micro‐ and nanopatterns.^6^ While multiple orthogonal cell–material interactions can be used to selectively bind cells to materials, these approaches do not capture the reversibility of cell adhesions and only provide limited spatiotemporal control.

The second challenge is the reversible control of cell adhesion to substrates both in space and time. Stimuli‐responsive materials mimic the dynamic nature of cell adhesion and can be used to alter cell adhesion with light, temperature, pH, and biochemical signals.[[qv: 2a]] In particular, light‐responsive cell–material interactions provide high spatiotemporal control. A general strategy for light‐controlled cell adhesion is caging RGD peptides, which are recognized by integrins, with photolabile nitrobenzyl caging groups that can be removed with UV light (350 nm) to render the RGD peptide active.^7^ Another strategy is to fuse RGD to azobenzene linkers, which undergo *trans* to *cis* isomerization upon UV light illumination, making the cell attachment and detachment reversible.^8^ The substantial disadvantage of these approaches is the exposure of cells to UV light, which is hazardous to cells. The direct exposure to UV light can be avoided by coupling photocleavable or switchable linkers to lanthanide‐doped upconversion nanoparticles, which can absorb NIR light (980 nm) and emit UV light.^9^ Although promising, the UV exposure of cells is still not avoidable. Further, strategies that rely on photo‐decaging are irreversible and the cell adhesion can only be altered once. Most importantly, none of these light‐responsive strategies provides independent control over multiple cell–material interactions in multicellular mixtures. The reason for this is the lack of photoswitchable cell adhesion ligands that specifically interact with different cells and that can be addressed orthogonally with different wavelengths of light. In this study, we show that the cell–material interactions of two different cell types can be orthogonally and reversibly controlled with blue and red light using photoswitchable proteins. Photoswitchable proteins have already been used as optogenetic building blocks to control many cellular processes including gene transcription,^10^ protein–protein interactions,^11^ cell signalling,^12^ organelle distribution,^13^ mechanotransduction,^14^ and viral gene delivery.^15^

In this study, we employed the blue light–dependent interaction between cryptochrome 2 (CRY2) and N‐truncated CRY‐interacting basic helix–loop–helix protein 1 (CIBN)[[qv: 12d]] as well as the red light–dependent interaction between phytochrome B (PhyB) and phytochrome interaction factor (6PIF6).[[qv: 12b]] CRY2 and PhyB change their conformations when exposed to blue light (480 nm) and red light (673 nm), respectively, and then bind to their specific interaction partners. While the CRY2/CIBN interaction only reverses in the dark, the PhyB/PIF6 interaction reverses in the dark and under far‐red light (750 nm). We expressed the photoswitchable proteins CRY2 or PhyB on the surfaces of living cells to turn on cell adhesion to substrates with the complementary interaction partners—CIBN or PIF6—under blue or red light, respectively, and reversibly turn them off in the dark (**Figure** **1**a).

**Figure 1:**
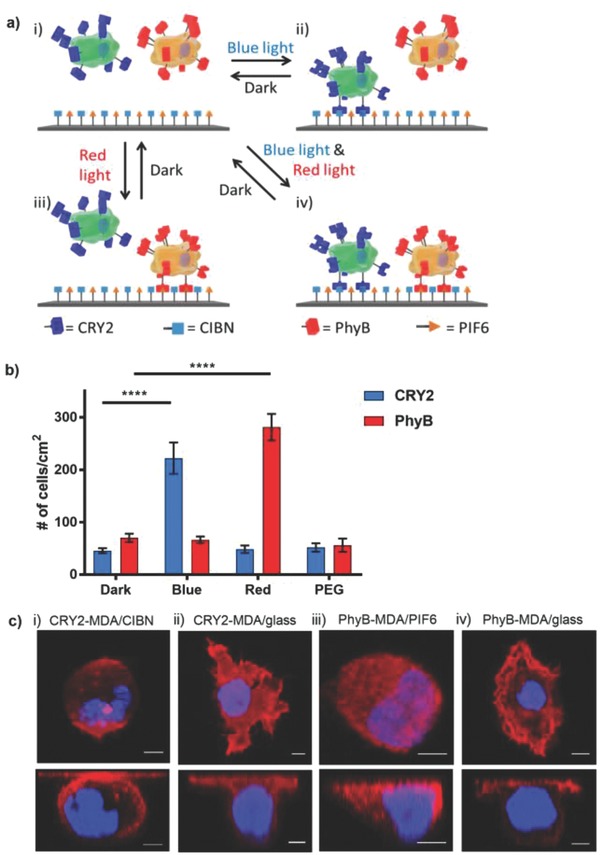
a) Cells that express CRY2 (green cell) or PhyB (orange cell) on their surfaces orthogonally bind to substrates with CIBN and PIF6 under blue or red light, respectively. i) In the dark, neither cell type binds to the substrate. ii) Under blue light, CRY2 changes conformation and CRY2 cells attach to CIBN‐functionalized substrates. iii) Under red light, PhyB changes conformation and PhyB cells attach to PIF6‐functionalized substrates. iv) Both CRY2 and PhyB cells bind to the substrate under co‐illumination with blue and red light. All these binding stages are reversible in the dark, and PhyB/PIF6 binding is also reversible under far‐red light. b) Quantification of light‐controlled cell–material interactions of CRY2‐MDA and PhyB‐MDA cells with CIBN‐ and PIF6‐functionalized substrates, respectively. The error bars are the standard error from nine technical replicates; unpaired *t*‐test is used as the statistical test (*p* value < 0.0001 (****)). c) Confocal images from the *z*‐axis (top) and side view (bottom) of CRY2‐MDA cells on i) CIBN‐functionalized substrates under blue light and ii) on glass, and PhyB‐MDA cells on iii) PIF6‐functionalized substrates under red light and iv) on glass. Red: actin; blue: nuclear DAPI stain. Scale bars are 5 µm.

These blue and red light–switchable cell–material interactions provide the desired high spatial and temporal control. Further, the interaction pairs CRY2/CIBN and PhyB/PIF6 switch with different wavelengths, which makes it possible to orthogonally address each cell type with a different color of light. The fact that these photoswitchable proteins respond to low‐intensity visible light enables noninvasive remote control without introducing light toxicity. These interactions require the modification of both the cell surface and the substrate with the appropriate proteins. The cell surface modification is genetically encoded and can be transfected into a cell type of choice, even into nonadherent cell types. These photoswitchable protein interaction pairs are very specific to each other and do not interfere with other biomolecules or one another. Moreover, these protein interactions are reversible in the dark and the PhyB/PIF6 interaction with far‐red light, which is an advantage for applications like cell sorting. As other synthetic cell adhesions, these light‐controlled interactions are not coupled to integrins and the associated cell signaling but can be combined with such at later stages by embedding cells in ECM.[[qv: 5a]] Overall, this approach provides high spatiotemporal control over two different cell–material interactions. They can be switched orthogonally with noninvasive blue or red light and are selective, bio‐orthogonal, and reversible.

In our approach to render cell–material interactions light responsive, we have to modify the cell surface and the substrate. To express CRY2 or PhyB on the surfaces of cells, we cloned CRY2 or PhyB into pDisplay plasmids (Figure 1a). pDisplay has an N‐terminal immunoglobulin (Ig) ĸ‐chain leader sequence to direct the protein to the secretory pathway, a fluorescent protein tag (mCherry for CRY2 and YFP (yellow fluorescent protein) for PhyB), and a C‐terminal transmembrane domain from the platelet‐derived growth factor receptor (PDGFR) to anchor the protein to the plasma membrane. Subsequently, we transfected MDA‐MB‐231 cells with one of the plasmids and generated the stable cell lines CRY2‐MDA and PhyB‐MDA. These two cell types continually expressed and displayed the light‐responsive proteins on the extracellular side of the cell, which we observed using the fluorescent protein tags in confocal microscopy images (Figure S1, Supporting Information). On the material side, we immobilized the complementary heterodimerization partners, CIBN or PIF6, on functionalized glass substrates. To achieve this, we first purified recombinantly expressed His6‐tagged CIBN and PIF6 and immobilized these proteins on glass substrates functionalized with a nonadhesive PEG (polyethylene glycol) coating with terminal Ni^2+^‐NTA‐groups (Figures S1 and S2, Supporting Information). We showed that the proteins are immobilized specifically through the His6‐tag binding to Ni^2+^‐NTA groups in QCM (quartz crystal microbalance) measurements, where the proteins stably bind to QCM crystal and only unbind in the presence of imidazole (Figure S3, Supporting Information). Next, we seeded CRY2‐MDA and PhyB‐MDA cells in the dark or under blue or red light on CIBN‐ and PIF6‐functionalized substrates, respectively. After 2 h incubation, we fixed the cells and stained their nuclei with DAPI to quantify the number of cells that bind to the substrate depending on the illumination conditions (Figure 1b). We observed that CRY2‐MDA cells bind better under blue light to the CIBN‐immobilized substrates, while PhyB‐MDA cells bind better under red light to PIF6‐immobilized substrates. However, neither CRY2‐MDA nor PhyB‐MDA cells bind to their complementary substrates in the dark. In fact, we used Ni^2+^‐NTA‐PEG‐coated substrates without immobilized proteins as negative controls and the numbers of CRY2‐MDA and PhyB‐MDA cells in the dark are comparable to those of the PEG control. We also tested CRY2‐MDA cell binding to CIBN‐immobilized substrates under red light and PhyB‐MDA cell binding to PIF6‐immobilized substrates under blue light to see if the other wavelength of light also activates cell–material interactions (Figure 1b). Our experiments showed each cell type binds to its substrate with its corresponding wavelength and that there is no cross‐talk between the two photoswitchable protein pairs. This provides us with two orthogonal cell–material interactions that can independently be addressed with two different wavelengths of visible light.

As the CRY2 and PhyB proteins we used are foreign photoswitchable cell adhesion molecules on the cell surface, we wanted to see if the photoswitchable interactions are strong enough to induce cell spreading. Additionally, we wanted to determine how they compare to native cell adhesion on glass. For this purpose, we seeded CRY2‐MDA cells on CIBN‐functionalized substrates under blue light and PhyB‐MDA cells on PIF6‐functionalized substrates under red light for 2 h. At the same time, we cultured CRY2‐MDA and PhyB‐MDA cells on bare glass substrates for the comparison. Subsequently, we stained the actin network with phalloidin‐TRITC to observe the cell spreading. First of all, confocal images of Cry2‐MDA and PhyB‐MDA cells cultured on their complementary substrates under blue and red light, respectively, showed that the cell membranes deform and the cells spread (Figure 1c, i, iii). The main difference between the light‐dependent cell adhesion and the adhesions on the glass substrates is the structure of the actin network. While, as expected, on the glass, the cells form an actin network (Figure 1c, ii, iv), the artificial photoswitchable cell adhesions did not connect to the actin network and actin remained homogenously distributed in the cell (Figure 1c, i, iii). Another measure of cell adhesion is the cell spreading area, which we analyzed using the actin staining. This analysis showed that there is no significant difference in the cell spreading area of cells that adhere to the substrates through the artificial photoswitchable cell receptors and the natural adhesions (Figure S4, Supporting Information). Overall, the photoswitchable cell–material interactions are similar to natural adhesions in terms of cell spreading, but they do not link to the actin cytoskeleton. This also implies that artificial cell interactions do not mediate the integrin‐dependent cellular signaling and cannot regulate integrin‐dependent cell functions such as migration and division.

Binding kinetics is an important aspect of cell interactions and cells generally require a couple of hours to allow them to attach, spread, and form mature focal adhesions on substrates. Given this, we evaluated the time that the blue and red light switchable cell–material interactions need to establish themselves by incubating cells on substrates with their complementary interaction partners under light and quantifying the number of cells on the substrate at different time points based on the nuclear DAPI staining. CRY2‐MDA cells adhered to CIBN‐immobilized substrates under blue light rapidly and, in 30 min, the maximum number of cells had bound. PhyB‐MDA cells bound to PIF6‐immobilized substrates under red light at a slower rate and the maximum number of cells attached after 60 min of incubation (**Figure** **2**a). Coincidentally, the numbers of CRY2‐MDA and PhyB‐MDA cells that adhere under blue and red light are almost the same for both the blue and red light–dependent adhesion.

**Figure 2:**
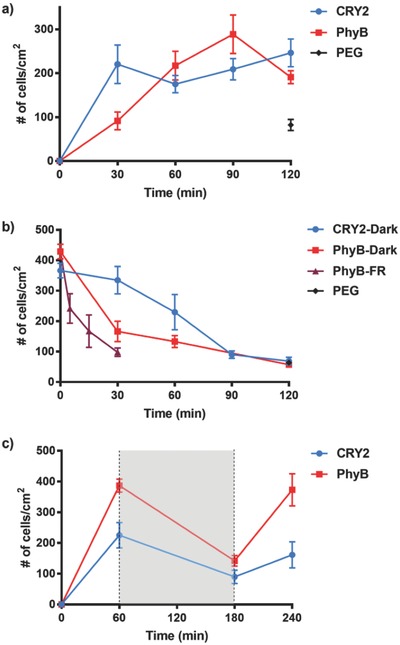
a) Adhesion kinetics of CRY2‐MDA cells on CIBN‐functionalized substrates under blue light and PhyB‐MDA cells on PIF6‐functionalized substrates under red light. b) Reversion kinetics of light‐controlled cell–material interactions. First, CRY2‐MDA cells attached to CIBN‐functionalized substrates under blue light and PhyB‐MDA cells attached to PIF6‐functionalized substrates under red light for 1 h. Then, CRY2‐MDA cells were moved into the dark and PhyB‐MDA cells into the dark or under far‐red (FR) light. c) Switching of light‐controlled cell–material interactions. First, CRY2‐MDA and PhyB‐MDA cells attached to CIBN‐ or PIF6‐functionalized substrates, respectively, for 1 h under light, then were left in the dark (shaded in gray) for 2 h and placed again for 1 h under light. Blue and red light were used for CRY2‐MDA and PhyB‐MDA cells, respectively. The error bars are the standard error of three biological replicates each done in three technical replicates.

As the CRY2/CIBN and the PhyB/PIF6 interactions are reversible in the dark, we expected that the cell–material interactions would reverse when illumination was stopped. Moreover, the PhyB/PIF6 interaction also reverses rapidly under far‐red illumination. In order to investigate if and how quickly the cells detach once the photoswitchable interactions are turned off, we first incubated CRY2‐MDA and PhyB‐MDA cells on their complementary substrates for 1 h under blue and red light, respectively. Subsequently, we placed the CRY2‐MDA cells in the dark and the PhyB‐MDA cells in the dark or under far‐red light illumination and quantified the number of cells on these substrates based on the nuclear DAPI staining at different time points (Figure 2b). Both the CRY2 and the PhyB expressing cells completely dissociated from their substrates within 2 h in the dark. When exposed to far‐red light, PhyB‐MDA cells detached even faster and completely dissociated after 30 min. These findings confirm that the cell–material interactions based on the blue and red light switchable proteins are reversible and that the PhyB/PIF6‐mediated interactions can be turned off orthogonally using far‐red light.

The CRY2/CIBN and the PhyB/PIF6 interactions are not only reversible but can also be switched on again under illumination. To demonstrate that cells can reattach with illumination, we incubated CRY2‐MDA and PhyB‐MDA cells on their complementary substrates first for 1 h under blue or red illumination, respectively, then kept them in the dark for 2 h, and finally placed them again under the same illumination for 1 h. As described above, we quantified the number of cells on the substrate at each stage based on the nuclear DAPI staining (Figure 2c). The number of cells that attach to the substrates increases after each illumination cycle of 1 h and decreases after 2 h in the dark for both the CRY2/CIBN‐ and PhyB/PIF6‐based cell interactions. This shows that both the CRY2/CIBN‐ and PhyB/PIF6‐mediated cell interactions can be switched on and off on demand.

The ultimate goal is to independently control the attachment of two different cell types in space and time (Figure 1a). This requires two orthogonal interaction pairs that can be switched using different wavelengths of light. To prove that the CRY2/CIBN‐ and the PhyB/PIF6‐based cell–material interactions fulfill these criteria, we co‐immobilized CIBN and PIF6 on substrates and investigated the attachment of mixtures of CRY2‐MDA and PhyB‐MDA cells depending on the illumination. In this experiment, we pre‐stained CRY2‐MDA cells with a red and PhyB‐MDA cells with a green fluorescence dye to distinguish between the two cell types and quantify them individually. When we co‐illuminated the cells with red and blue light, we observed that both CRY2‐MDA and PhyB‐MDA cells equally bound to the substrate (**Figure** **3**a,b). On the other hand, neither of the cell types bound to the substrate in the dark and the numbers of cells on these substrates were comparable to a substrate with just a PEG coating. We could also induce the attachment of just one cell type at a time depending on the wavelength of light used; CRY2‐MDA cells attached to the substrate under blue light and PhyB‐MDA cells attached under red light.

**Figure 3:**
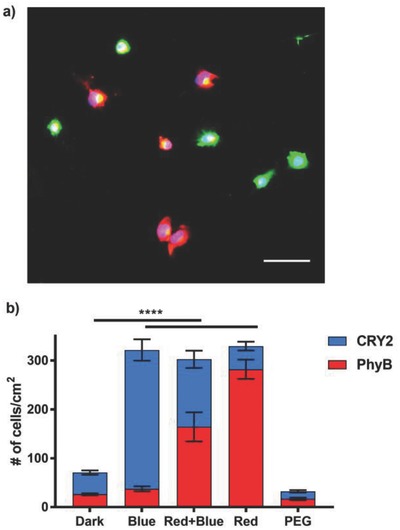
Orthogonal blue and red light switchable cell adhesions. a) Fluorescence image of CRY2‐MDA (in red) and PhyB‐MDA (in green) cells on CIBN‐ and PIF6‐co‐immobilized substrates under co‐illumination with blue and red light. Both cell types equally adhere to the substrate. Scale bar: 75 µm. b) Quantification of the number of CRY2‐MDA and PhyB‐MDA cells in the dark, under red and blue co‐illumination, and only red or only blue light illumination. The contribution of CRY2‐MDA cells is shaded in blue and the contribution of the PhyB‐MDA cells is shaded in red. The number of cells was calculated from red (CRY2‐MDA) and green (PhyB‐MDA) fluorescence channels. Average of three biological replicates each done in three technical replicates; unpaired *t*‐test is used as the statistical test (*p* value < 0.0001 (****)).

We have developed two photoswitchable cell–material interactions that are orthogonal to each other and respond to two different wavelengths of visible light. This enables us to induce cell adhesions independently of one specific cell type at a time by using either blue or red light. The fact that these photoswitchable cell–material interactions are reversible makes it possible to dynamically attach and detach a specific cell type. Unlike previous light‐responsive cell adhesions, which respond to UV light, these interactions respond to low‐intensity visible light, which is noninvasive to cells. These visible light‐responsive cell–material interactions only capture the physical binding of different cell types to materials and, in the future versions, that couple to integrin‐dependent cell signaling and functions could be developed. Overall, these orthogonal blue and red light switchable cell–material interactions will open the way for future developments in the development of multicellular systems, where dynamic and high spatiotemporal control over multiple cell–material interactions is required.

## Conflict of Interest

The authors declare no conflict of interest.

## Supporting information

Supplementary
