# Supplementary material for: Independent Control over Multiple Cell Types in Space and Time Using Orthogonal Blue and Red Light Switchable Cell Interactions
Source: Adv Sci (Weinh). 2018 Jun 17;5(8):1800446. doi: 10.1002/advs.201800446 (PMC6097145; doi:10.1002/advs.201800446)
Supplement: Supplementary file 1 — Supplementary [file ADVS-5-1800446-s001.pdf]

Copyright WILEY-VCH Verlag GmbH & Co. KGaA, 69469 Weinheim, Germany, 2018.

Supporting Information

for *Adv. Sci.,* DOI: 10.1002/advs.201800446

Independent Control over Multiple Cell Types in Space and Time Using Orthogonal Blue and Red Light Switchable Cell Interactions

*Simge G. Yüz, Julia Ricken, and Seraphine V. Wegner**

Copyright WILEY-VCH Verlag GmbH & Co. KGaA, 69469 Weinheim, Germany, 2016.

Supporting Information

Independent Control over Multiple Cell Types in Space and Time using Orthogonal Blue and Red Light Switchable Cell Interactions

*Simge G. Yüz, Julia Ricken and Seraphine V. Wegner**

Experimental Section

Materials. pCRY2FL(deltaNLS)-mCherryN1 and pCIBN(deltaNLS)-pmGFP were a gift from Chandra Tucker (Addgene plasmid # 26871 and 26867), pAL149 and pAL175 were a gift from Christopher Voigt (Addgene plasmid # 22275 and 22276) and pDisplay-AP-CFP-TM was a gift from Alice Ting (Addgene plasmid # 20861). All enzymes were purchased from New England Biolabs and all chemicals from Sigma Aldrich unless indicated). All cell culture reagents are purchased from Gibco by Life Technologies distributed by Thermo Fisher Scientific unless indicated. MDA-MB-231 cells were purchased from ATCC.

Cloning. Genes encoding CRY2-mCherry, PhyB and PIF6 are PCR-amplified using gene-specific primers from the plasmids pCRY2FL(deltaNLS)-mCherryN1 (Addgene plasmid # 26871),[1] pAL149 (Addgene plasmid # 22275)[2] and pAL175 (Addgene plasmid # 22276),[2] respectively. The PCR products CRY2-mCherry and PhyB are then ligated into pDisplay (Invitrogen) between the cloning sites SacII/SalI and SacII/XmaI to yield pDisplay-CRY2-mCherry, and pDisplay-PhyB, respectively. The gene encoding for YFP is PCR amplified from pAL175 (Addgene plasmid # 22276)[2] and inserted between SacII/PstI of pDisplay-PhyB to yield pDisplay-PhyB-YFP. The PCR product PIF6 is ligated into pDisplay-AP-CFP-TM (Addgene plasmid # 20861)[3] between the cloning sites BglII/AscI to yield pDisplay-PIF6-CFP. CIBN-GFP and PIF6-CFP are PCR-amplified from pCIBN(deltaNLS)-pmGFP (Addgene plasmid # 26867)[1] and pDisplay-PIF6-CFP plasmids and the PCR products are ligated into pET21b (Novagen) between the cloning sites NdeI/XhoI with a C-terminal His6-

tag. After the sequences are confirmed by sequencing, pDisplay-CRY2-mCherry and pDisplay-PhyB-YFP are used to produce stable cell lines and pET21b-CIBN-GFP and pET21b-PIF6-CFP are used for protein expression in *E.coli*. Abbreviations: CRY2: cryptochrome 2, CIBN: N-truncated Cry-interacting basic helix-loop-helix protein 1, PhyB: phytochrome B, PIF6: Phytochrome interacting factor 6, GFP: green fluorescent protein, CFP: Cyan fluorescent protein, YFP: Yellow fluorescent protein.

Protein Purification. pET21b-CIBN-GFP and pET21b-PIF6-CFP are transformed into *E. coli* BL21 (New England Biolabs). The bacteria are grown at 37 °C and 220 rpm in 1 l LB medium with 50 µg/ml ampicillin until they reach a density of OD_{600} = 0.4-0.6. Protein expression is induced with 0.5 mM IPTG and then the bacterial culture is incubated at 16 °C overnight. The next day bacteria are harvested by centrifugation (6000 g, 8 min), suspended in 20 ml Buffer A (300 mM NaCl and 50 mM TRIS, pH 7.4) supplemented with 1.25 mM PMSF and lysed by sonication. The lysate is clarified by centrifugation and the protein in the supernatant is purified by using a Ni^{2+}-NTA His-trap column and followed by gel filtration (GE Healthcare Europe GmbH). The protein purity is verified by SDS-PAGE (**Figure S1b**).

Substrate functionalization and protein immobilization. The glass substrates are coated with a PEG (polyethylene glycol) as previously described.[4] Shortly, 20 mm x 20 mm glass cover slips (Carl Roth GmbH & Co. KG) are cleaned in piranha solution (3:1 H_2SO_4: H_2O_2) for 1 hr at room temperature, rinsed thoroughly with water and dried with a nitrogen stream. The substrates are immersed in a solution of 0.25 mM PEG-N_3 (PEG 3000 with a terminal azide and a terminal triethoxysilane group) (Iris Biotech GmbH) and 25 µM triethylamine in dry toluene at 78 °C overnight under a nitrogen atmosphere. Subsequently, the substrates are sonicated in ethyl acetate for 5 min, in methanol for 5 min and dried in a stream of nitrogen. For click reaction, the PEGylated substrates are incubated with a freshly prepared aqueous solution of 100 mM *L*-ascorbic acid, 150 µM NTA-alkyne (NTA: nitrilotriacetic acid),[5] 100 mM TRIS pH 9.5 and 1 mM $CuSO_4$ for 2 hours in a moisture chamber (Figure S2).

Afterwards, the substrates are washed twice with Buffer A, once with 50 mM EDTA and twice with Buffer A. Then, the substrates are incubated with 100 mM $NiCl_2$ to coordinate Ni^{2+} to the NTA groups and washed with Buffer A. Finally, the Ni^{2+}-NTA functionalized substrates are incubated with 10 µM His6-tagged protein for 30 min at room temperature.

QCM-D (Quartz crystal microbalance with dissipation monitoring) measurements. SiO_2 QCM-D sensors (Q-sense) are cleaned with an aqueous solution of SDS (sodium dodecyl sulfate) (2 % w/v) overnight, washed thoroughly with water and dried in a nitrogen stream. Subsequently, the SiO_2 sensors are cleaned and activated with oxygen plasma (0.4 mbar, 150 W, 45 min) and functionalized with a PEG coating with terminal Ni^{2+}-NTA groups as described above. All QCM-D measurements are performed on a Qsense-Explorer at a flow rate of 100 µl/min. The functionalized crystals are washed with Buffer A until a baseline is reached, before 10 µM His6-tagged proteins are washed over the crystals. Then, the unbound proteins are washed off with Buffer A and the specifically bound proteins are eluted with Buffer A supplemented with 250 mM imidazole).

Generation of stable cell lines. All cells are cultured in Dulbecco's Modified Eagle Medium (DMEM)/F12 (1:1) supplemented with 10 % FBS (fetal bovine serum) and 1 % P/S (penicillin/streptomycin) at 37 °C in 5 % CO_2 atmosphere. MDA-MB-231 cells are seeded on polystyrene plates at a density of 6.25 x 10^4 cells/cm^2 and grown overnight. To generate the stable cells lines, the cells are transfected with pDisplay-CRY2-mCherry or pDisplay-PhyB-YFP plasmids by using Lipofectamine LTX reagent (Thermo Fisher Scientific) following the instructions provided by the manufacturer. After 24 hours, cells are selected over 0.9 mg/mL geneticin to yield CRY2-MDA and PhyB-MDA cell. The cells are cultured in the presence of 0.9 mg/ml geneticin for all the further experiments. The expression of CRY2-mCherry and PhyB-YFP in the stable cell lines is observed with a confocal microscope (LSM 880, Carl Zeiss Microscopy GmbH, Jena, Germany) equipped with UV diode (405 nm), Ar laser (488 nm), HeNe laser (633 nm) and a 63x oil-immersion objective.

Light dependent cell adhesion. CRY2-MDA and PhyB-MDA cells are detached from the culturing flasks by incubation in the presence of accutase for 7 min at 37°C and then are seeded on CIBN and PIF6 functionalized glass substrates in phenol-red free DMEM/F12 (1:1) medium supplemented with 0.9 mg/ml geneticin at a density of 4000 cells/cm^2 and incubated in dark, under blue light (471 nm, 80 µW/cm^2) or red light (673 nm, 55 µW/cm^2) for 2 hours. In all experiments where PhyB-MDA cells are used the medium is further supplemented with 5 µM PCB (phycocyanobilin), which is not available in orthologous systems and is extracted from the cyanobacterium Spirulina powder following the protocol from Müller *et al.* [6] A substrate just with a PEG coating and without immobilized protein is used as a negative control. After incubation, cells are fixed with 4% PFA (paraformaldehyde) for 15 min at 37 °C under 5 % CO_2 atmosphere in dark, under blue or red light. After fixation, cells are washed with PBS (phosphate buffer saline), permeabilized with 0.1 % Triton X-100 for 5 min, stained for actin with 0.5 µg/mL TRITC conjugated phalloidin for 30 min and mounted with mowiole 4-88 (Carl Roth GmbH & Co. KG) containing 1 µg/mL DAPI. The adhesion kinetics for CRY2-MDA and PhyB-MDA cells are measured similarly by incubating the cells on their complementary substrates under blue and red light, respectively, and fixing a sample at each time point. For reversibility experiments the cells first adhered to their complementary substrate under appropriate illumination before placing them in the dark and under far-red light (750 nm, 50 µW/cm^2) for the PhyB-MDA cells. A sample is fixed at each time point. For repeated switching of the adhesions, the cells first adhered to their complementary substrate under appropriate illumination for 1 hr. Then the cells are incubated in the dark for 2 hrs. Subsequently, the cells are incubated under the corresponding light illumination for 1 hr. At each switching point, three substrates are fixed and stained with DAPI as described above. To demonstrate the independent activation of the blue and red light switchable cell adhesion, CRY2-MDA and PhyB-MDA cells are pre-stained with the lipophilic tracers DiI (1,1' -dioctadecyl-3,3,3'3'-tetramethylindocarbocyanine perchlorate) and DiO (3,3' -

Dioctadecyloxacarbocyanine Perchlorate) (Sigma Aldrich), respectively by adding these dyes in the cell culture medium at a final concentration of 6 µM DiO and 3 µM DiI and incubating them overnight. Alternatively, CRY2-MDA and PhyB-MDA cells were stained with Cell Mask Orange and Cell Mask Deep Red (Thermo Fischer Scientific), respectively, following the manufacturers protocol. PIF6 and CIBN are co-immobilized on the NTA-functionalized glass substrates by incubating a PEG coated substrate with terminal Ni^{2+}-NTA groups with 10 µM PIF6-CFP-His6 and 10 µM CIBN-GFP-His6 in Buffer A for 30 min. The prestained CRY2-MDA and PhyB-MDA cells are mixed in a 1:1 ratio and seeded at a total density of 8000 cells/cm^2. The cells are incubated in dark, under red and blue light (coillumination), just under blue light or just under red light for 2 hours. Subsequently, the cells are fixed and mounted with mowiole 4-88 containing 1 µg/mL DAPI. Each experiment was performed in three biological replicates with three technical replicates each (n=9).

Image analysis. All images for image analysis are acquired with an upright fluorescence microscope (Leica DM6000B) equipped with a 10x air lens objective and by tile scanning of an area of 1 cm^2 for each sample. To measure the number of cells on the substrate fluorescence images are acquired in the DAPI channel for all samples. To quantify the number of CRY2-MDA and PhyB-MDA cells images are acquired in the GFP and TRTIC channels for samples with pre-stained CRY2-MDA (DiI, red fluorescence, TRITC channel) and PhyB-MDA (DiO, GFP channel) cells. All images are analyzed with ImageJ (ImageJ 1.51f) and the number of cells is determined using the analyze particle tool (The area of the objects is set to be larger than 50 μm^2 and smaller than 5000 μm^2). All experiments are done in technical triplicates with 3 replicates in each set. The error is defined as the standard error. The Mann-Whitney test is performed for statistical analysis in GraphPad Prism and statistical significance is indicated on the figures. The phalloidin-TRITC staining is used to evaluate the cell spreading area and images are acquired as described above. The Cell Outliner tool in ImageJ (ImageJ 1.51f) is used measure the cell spreading area of 30 randomly selected cells.

2. References

[1] M. J. Kennedy, R. M. Hughes, L. A. Peteya, J. W. Schwartz, M. D. Ehlers, C. L. Tucker, *Nat. Methods* **2010**, *7*, 973-975.

[2] A. Levskaya, O. D. Weiner, W. A. Lim, C. A. Voigt, *Nature* **2009**, *461*, 997-1001

[3] M. Howarth, K. Takao, Y. Hayashi, A.Y. Ting, *Proc. Natl. Acad. Sci. U.S.A.* ***2005**, 102, 7583-7588.*

[4] F. C. Schenk, H. Boehm, J. P. Spatz, S. V. Wegner, *Langmuir* **2014**, *30*, 6897-6905.

[5] Y. Li, J. C. Niehaus, Y. Chen, H. Fuchs, A. Studer, H. –J. Galla, L. Chi, *Soft Matter* **2011**, *7*, 861-863.

[6] K. Muller, M. D. Zurbriggen, W. Weber, *Nat. Protoc.* **2014**, *9*, 622-632.

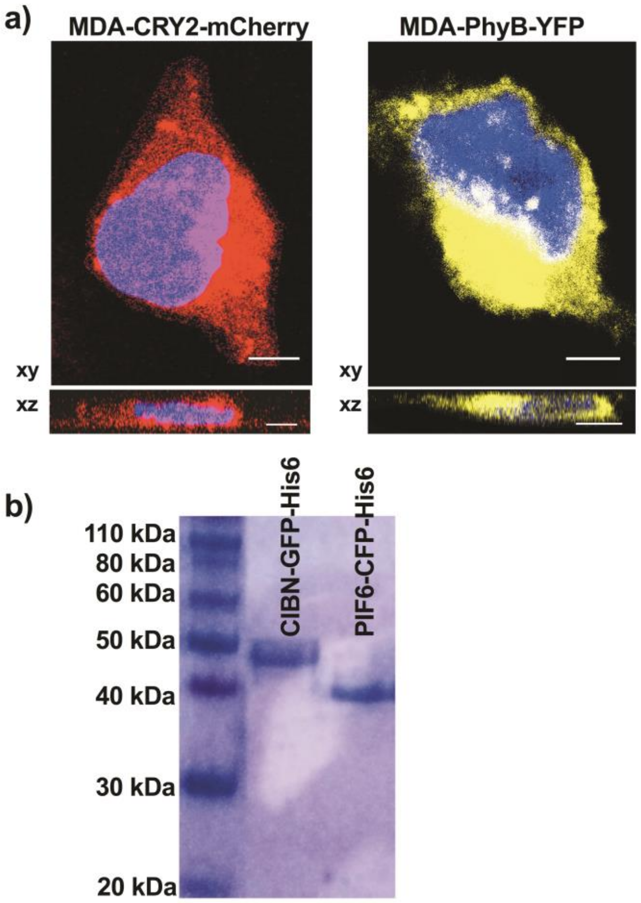

Figure S1. a) Confocal images of stable cell lines CRY2-MDA and PhyB-MDA. CRY2 is fused to mCherry and PhyB is fused to YFP. Blue: DAPI, red: mCherry, yellow: YFP. Scale bar: 5 µm. **b)** SDS-PAGE of purified proteins. PIF6-CFP-His6: 40 kDa and CIBN-GFP-His6: 50 kDa.

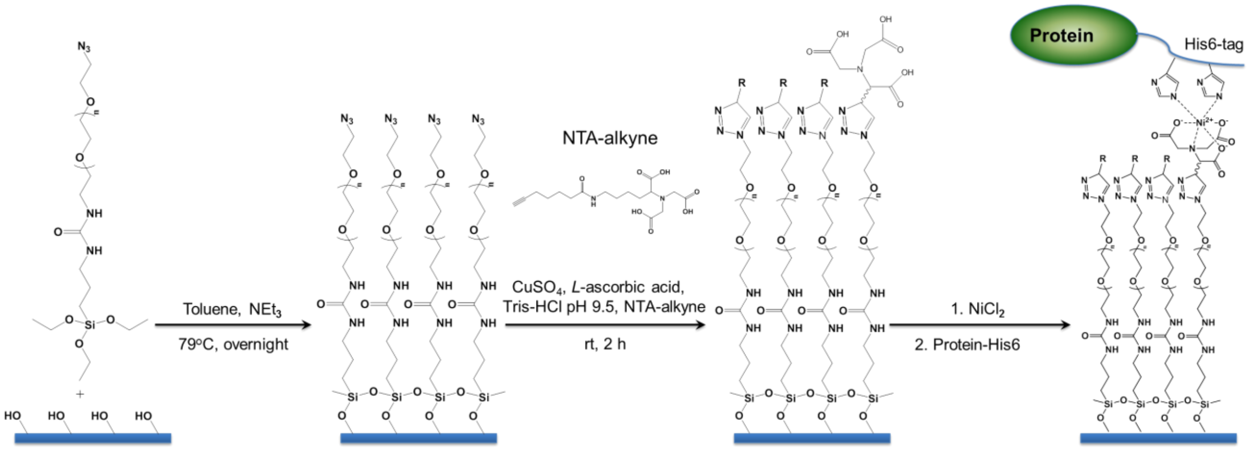

Figure S2. Functionalization scheme of glass substrates with proteins. Glass substrates are coated with a nonadhesive PEG3000 with a terminal azide group (PEG-N_3) overnight in toluene at 78°C. Then, NTA-alkyne group is clicked on the substrate and loaded with Ni^{2+}. Finally, the His6-tagged proteins are immobilized on the substrate through His6-Ni^{2+}-NTA binding.

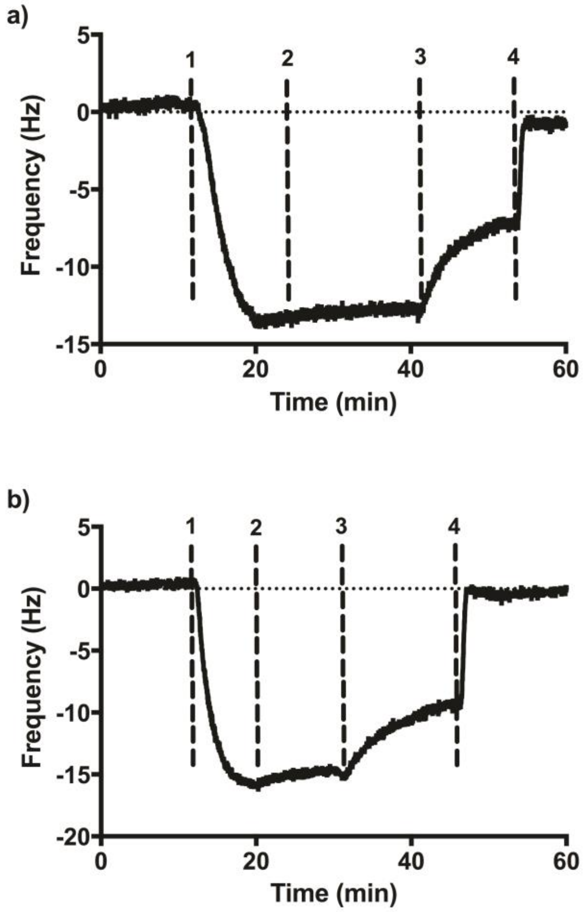

Figure S3. QCM measurements showing specific protein binding of the His6-tagged proteins to the Ni^{2+}-NTA functionalized PEG substrates. **a)** CIBN-GFP-His6 and **b)** PIF6-CFP-His6. Following components in Buffer A are washed over the QCM crystal at indicated time points. 1) 10 µM His6-tagged protein, 2) Buffer A, 3) 250 mM imidazole and 4) Buffer A. Unspecific protein binding and excess of protein is washed off with Buffer A at time point 2. The specific interaction between Ni^{2+}-NTA and His6-tagged proteins is reversed in the presence of imidazole.

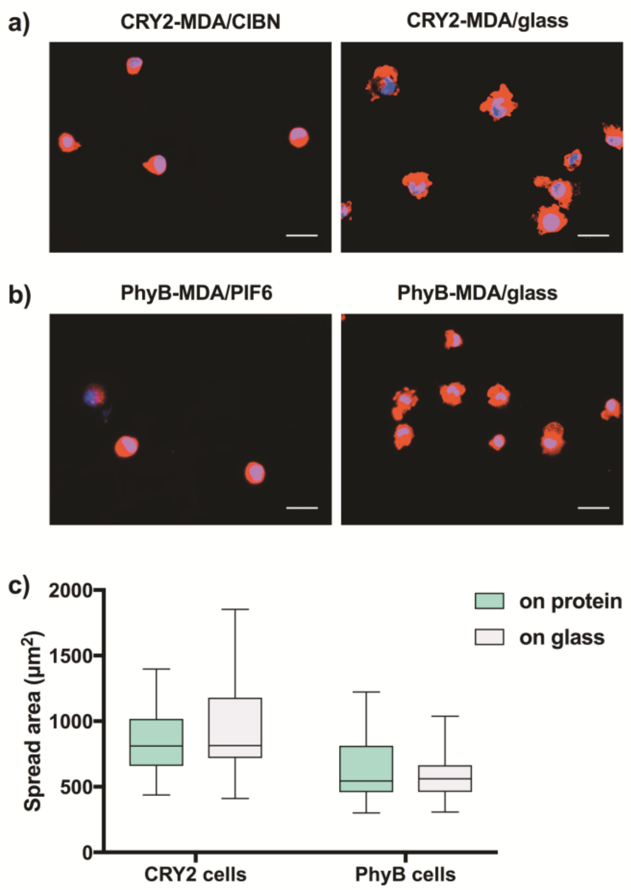

Figure S4. Cell spreading of CRY2-MDA and PhyB-MDA cells on CIBN and PIF6 functionalized substrates, respectively. Fluorescence images of **a)** CRY2-MDA cells on CIBN functionalized substrates under blue light and on glass, and **b)** PhyB-MDA cells on PIF6 functionalized substrates under red light and on glass. Red: Phalloidin-TRITC, blue: DAPI. Scale bar: 50 µm. **c)** Quantification of cell spreading area of CRY2-MDA and PhyB-MDA cells on their commentary protein substrate under illumination and on glass. The cells are stained for actin with phalloidin-TRITC and the spreading area is calculated for 30 randomly selected cells. For both cell types, the spreading area is the same no matter if they adhere through the photoswitchable cell adhesions or integrin mediated adhesion on glass.
